# A Computational Model of the LGI1 Protein Suggests a Common Binding Site for ADAM Proteins

**DOI:** 10.1371/journal.pone.0018142

**Published:** 2011-03-29

**Authors:** Emanuela Leonardi, Simonetta Andreazza, Stefano Vanin, Giorgia Busolin, Carlo Nobile, Silvio C. E. Tosatto

**Affiliations:** 1 Department of Biology, University of Padova, Padova, Italy; 2 School of Applied Science, University of Huddersfield, Huddersfield, United Kingdom; 3 Institute of Neurosciences, Consiglio Nazionale delle Ricerche (CNR), Padova, Italy; German Cancer Research Center, Germany

## Abstract

Mutations of human leucine-rich glioma inactivated (*LGI1*) gene encoding the epitempin protein cause autosomal dominant temporal lateral epilepsy (ADTLE), a rare familial partial epileptic syndrome. The *LGI1* gene seems to have a role on the transmission of neuronal messages but the exact molecular mechanism remains unclear. In contrast to other genes involved in epileptic disorders, epitempin shows no homology with known ion channel genes but contains two domains, composed of repeated structural units, known to mediate protein-protein interactions.

A three dimensional *in silico* model of the two epitempin domains was built to predict the structure-function relationship and propose a functional model integrating previous experimental findings. Conserved and electrostatic charged regions of the model surface suggest a possible arrangement between the two domains and identifies a possible ADAM protein binding site in the β-propeller domain and another protein binding site in the leucine-rich repeat domain. The functional model indicates that epitempin could mediate the interaction between proteins localized to different synaptic sides in a static way, by forming a dimer, or in a dynamic way, by binding proteins at different times.

The model was also used to predict effects of known disease-causing missense mutations. Most of the variants are predicted to alter protein folding while several other map to functional surface regions. In agreement with experimental evidence, this suggests that non-secreted LGI1 mutants could be retained within the cell by quality control mechanisms or by altering interactions required for the secretion process.

## Introduction

The human leucine rich, glioma inactivated 1 (*LGI1*; GeneID 9211; MIM# 604619) gene has been linked to two different clinical phenotypes: malignant progression of glioma and autosomal dominant lateral temporal epilepsy (ADLTE; MIM# 600512), a rare familial partial epilepsy syndrome. This gene has been shown to be frequently downregulated in malignant gliomas and to regulate invasiveness of some glioma cell lines [1] by driving the expression of matrix metalloproteinases through the ERK 1/2 pathway. These findings suggest that *LGI1* may serve as a tumor metastasis suppressor gene [2].

ADTLE is an inherited epileptic syndrome characterized by focal seizures with predominant auditory symptoms likely originating from the lateral temporal lobe cortex [3], [4]. Mutations causing ADLTE were identified in the *LGI1* gene by positional cloning [5], [6]. To date, over 25 mutations have been reported, resulting in either protein truncation or single amino acid substitutions [7], but about half of the ADLTE families have no *LGI1* mutations [3]. *LGI1* is mainly expressed in neurons [6], [8] and shows no similarity to known ion channels. The predicted structure of the LGI1 protein comprises, starting from the N-terminal end, a signal peptide, four leucine-rich repeats (LRR) flanked on both sides by conserved cysteine clusters [9], and seven copies of a repeat of about 45 residues, named EPTP [10] or EAR [11], probably forming a β-propeller structural domain [12]. Both LRR and β-propeller domains mediate protein-protein interactions, each motif defining a distinct family of proteins [12], [13].

Several different functions and molecular partners have been attributed to LGI1. A recent study provided evidence that LGI1 is associated with a post-synaptic complex containing PSD95 and ADAM22, a receptor associated with the post-synaptic membrane [14]. Through specific binding to ADAM22, LGI1 was shown to participate in the control of synaptic strength at excitatory synapses, whose malfunction may result in epilepsy [14]. Mouse models developed more recently have implicated LGI1 in neuronal maturation processes. In one study, it was shown that LGI1 affects postnatal maturation of glutamatergic synapses, a process involving ADAM22, and mediates dendrite pruning so that LGI1 mutations would result in persistence of immature, untrimmed, dendritic arbor [15]. On the other hand, another study showed that LGI1 preferentially interacts with ADAM23 and through this receptor, which is not located at postsynaptic density, stimulates neurite outgrowth *in vitro* and dendritic arborisation *in vivo* [16]. Finally, analysis of *LGI1* knock-out and transgenic mice suggested that LGI1 may act as a trans-synaptic protein connecting the pre-synaptic ADAM23 with the post-synaptic ADAM22 receptors [17].

To help understand the three dimensional (3D) conformation of LGI1, its binding properties, and ultimately its function(s), we developed an *in silico* model of the protein structure and analysed the amino acid sequence of the LRR and β-propeller LGI1 domains as well as their phylogenetic relationship. The models were used to assess the significance of known missense mutations. Analysis of possible interaction mechanisms with other proteins suggests a conserved common binding site for members of the ADAM protein family.

## Materials and Methods

### Sequence feature analysis

We employed an integrative bioinformatics approach combining sequence and domain database searches with the consensus from predictions of protein structural features. The LGI1 sequence (accession code: O95970) was downloaded from the SwissProt/TrEMBL database [18]. Homologous sequences were retrieved and selected with BLAST [19] from the SwissProt database using standard parameters and visualized using Jalview [20] and ESPript [21]. The secondary structure of LGI1 was predicted using the *consensus* method [22]. Prediction of intrinsic disorder was performed using Spritz [23] and the presence of signal peptides assessed with SignalP [24]. Repetita [25] was used to predict repeat periodicities.

### Phylogenetic analysis

In order to reconstruct the phylogeny of the LGIs, 105 vertebrate and one branchiostomid epitempin sequences have been automatically extracted from the available databases using BLAST [19] searches. Full-length amino acid sequences have been recovered from the corresponding nucleotide mRNA or genomic sequences. Multiple alignment was constructed with CLUSTALW [26]. The final alignment has been manually refined at the variable N-terminus and used in the subsequent analysis.

A preliminary quartet puzzling analysis has been performed with the Treepuzzle program [27], [28] to test whether a phylogenic approach could be applied to the original data set. Phylogenic studies have been performed according to the maximum likelihood (ML) with the PHYML 2.4 program [29]. The JTT substitution matrix [30] was used during reconstruction, whereas site heterogeneity was modeled with a four-category Γ distribution. Nonparametric bootstrap resampling (BT) [31] was performed with 1,000 replicas to test the robustness of the tree topology. The phylogenetic tree was visualized with the Fig Tree 1.1.1 program (http://tree.bio.ed.ac.uk/software/figtree/).

### Alignment construction

Structural templates for the two LGI1 domains were found using MANIFOLD [32] and MetaServer [33]. Initial alignments were generated through systematic parameter variation from an ensemble of similar alternatives [34]. Given the problematic nature of repeated sequences, the best initial alignment was used as a starting point only. Manual refinement consisted in a method similar to ABRA [35] and Kajava's method [36], with knowledge about the approximate location and number of repeats serving to identify the true repeat boundaries. Knowledge of key residues and secondary structure was used to anchor the aligned repeats.

### Molecular modeling

Models for the two LGI1 domains were constructed using the HOMER server (*URL:* http://protein.bio.unipd.it/homer/). The server uses the conserved parts of the structure to generate a raw model, which is then completed by modeling the divergent regions with LOBO, a fast divide and conquer method [37]. Side chains are placed with SCWRL3 [38] and the energy evaluated with FRST [39]. The final models were subjected to a short steepest descent energy minimization with GROMACS [40] to remove energy hotspots before calculating the electrostatic surface with APBS [41]. Evaluation of model quality was performed with QMEAN [42], [43]. The structure is visualized using PyMOL (DeLano Scientific, URL: http://pymol.sourceforge.net/). Position-specific conservation scores for each amino acid were calculated with ConSurf [44].

### Mutation analysis

Amino acid substitutions have been mapped on the LRR and EPTP domain models and their position evaluated by manual inspection. Four computational methods were used to predict the stability change of the structure caused by these mutations. While I-Mutant 2.0 [45] and MuPro [46] both utilize support vector machines or neural networks to predict the effect of the substitution on protein stability, Eris [47] and PoPMuSiC v2.0 [48] calculate mutational free energy changes of the protein based on its 3D structure.

## Results and Discussion

Given the fragmented knowledge present in the literature, we performed a full analysis of the LGI protein family starting from the protein sequence. In the following, we will address each step from phylogeny to sequence and structural analysis all the way to new functional hypotheses.

### Phylogenetic analysis

**Figure 1:**
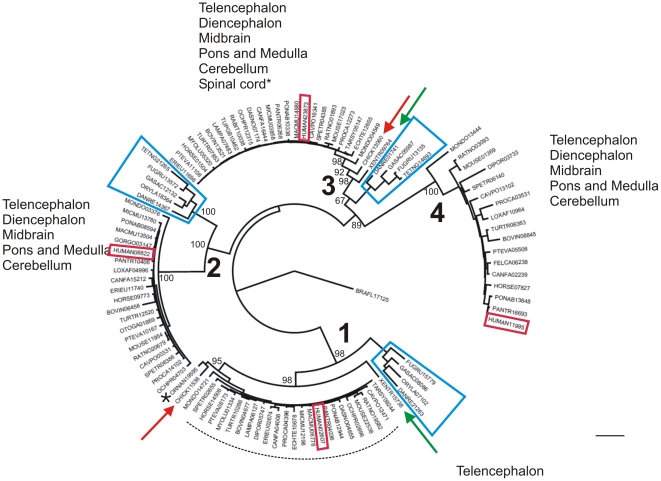
Evolutionary relationship among the LGI vertebrate amino acid sequences. The figure shows the best likelihood tree (−lnL = −21148.01332) obtained using the PHYML program. The length of the branches represents the number of reconstructed change of state over all sites (bar represents 0.2 substitutions per site), bootstrap values are reported at the nodes. Blue squares indicate the fish sequences whereas the green and red arrows respectively the amphibian and bird sequences. An asterisk indicates the *Ornithorhynchus anatinus* protein.

The phylogenetic reconstruction was performed using 105 Vertebrate (Chordata; Chraniata) sequences. An additional sequence of *Branchiostoma floridae* (Chordata; Cephalochordata) has been included in the analysis. The obtained reconstruction reported in Figure 1 highlights the presence of 4 groups, named 1, 2, 3 and 4. The distribution pattern of LGI family transcripts in the adult mouse brain [49] highlights the tissue specificity of group 1 (see Figure 1).

Group 1, 2 and 3 present the fish sequences (blue squares) in a basal position, followed in group 1 and 3 by amphibian and bird sequences (red and green arrows). The mammalian sequences present an apical position in all the groups. The *Ornithorhynchus anatinus* protein shares a common node with chicken in group 1 and both are basal to the other mammalians. The phylogeny of LGI1 reveals an early duplication of the gene followed by two other independent duplications as already reported by Gu et al [50], but, in contrast to these authors, the phylogeny obtained with a larger dataset indicates a closer relationship between the LGI3 and LGI4 sequences as opposed to LGI1 and LGI4.

### Sequence domain organization

**Figure 2:**
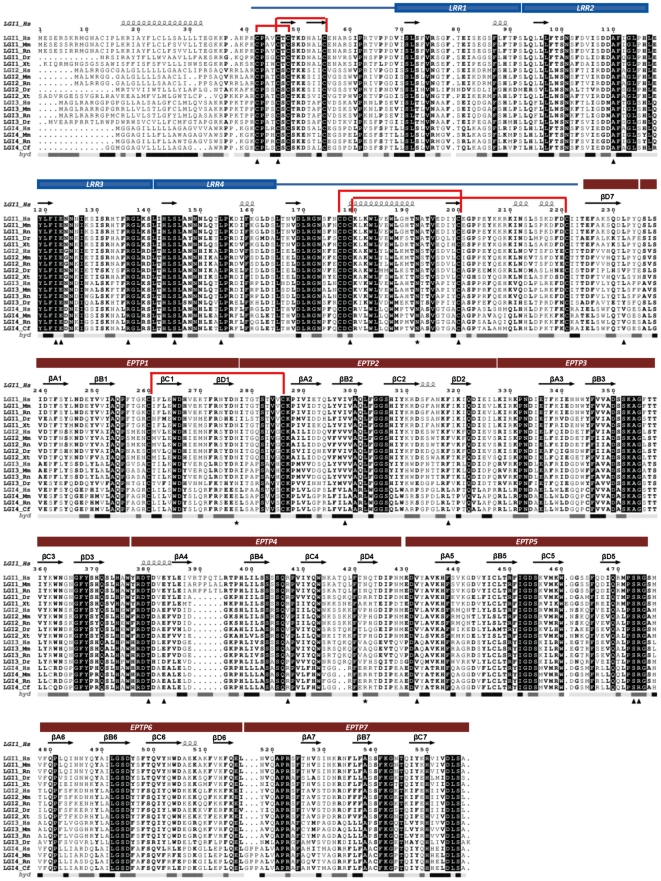
Alignment of LGI family members and domain organization. Multiple alignment of representative homologs in the LGI family. Species are abbreviated as follows: Hs = *Homo sapiens*; Mm = *Mus musculus*; Rn = *Rattus norvegicus*; Dr = *Danio rerio*; Xt = *Xenopus tropicalis*; Cf = *Canis familiaris*. The LGI1 domains and secondary structure are shown on the top part. Missense mutations analyzed in this paper (triangles) and putative glycosylation sites (stars) are indicated on the bottom of the alignment. Red lines are used to connect cysteine residues that form disulphide bridges in the structural model. *acc*: accessibility level from DSSP (black = high and white = low).

We defined boundaries of each domain in the LGI1 sequence (Figure 2). The first 35 N-terminal residues contain the signal peptide responsible for its secretion. A cleavage site is also predicted by SignalP in this region. The N-terminal part of the protein from residues 41 to 243 has about 30% sequence identity with LRR domain family proteins, while the C-terminal region between residues 245–552 contains the EPTP repeats. The two domains are also present in all human LGI proteins (LGI1, LGI2, LGI3, LGI4) and conserved across orthologs (Figure 2). Since a structure of LGI1 is not available, a structural analysis was conducted separately for the two domains as they have different characteristics.

### Homology modeling of LRR domain and sequence to structure mapping

**Figure 3:**
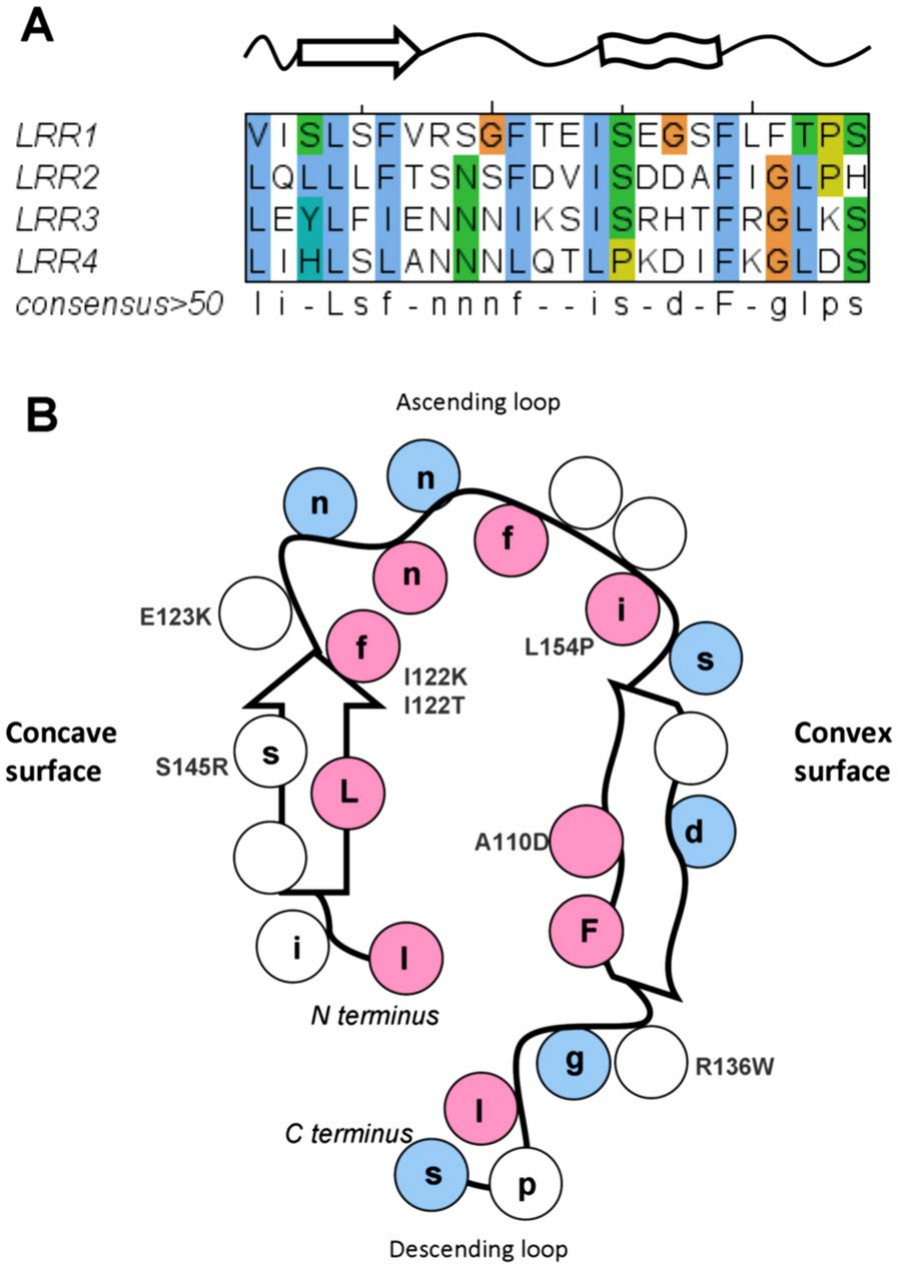
LRR repeat overview. A. Consensus sequence repeat pattern of the LRR domain. Secondary structure is drawn on the top part of the alignment: an arrow represents the β-strand and a ribbon the α-helix connected by curved lines (loops). B. Schematic diagram of repetitive structural units in LGI1 protein. Conserved positions of the consensus pattern are reported on the diagram. Coloured pink spheres for buried residues and blue spheres for exposed residues.

**Figure 4:**
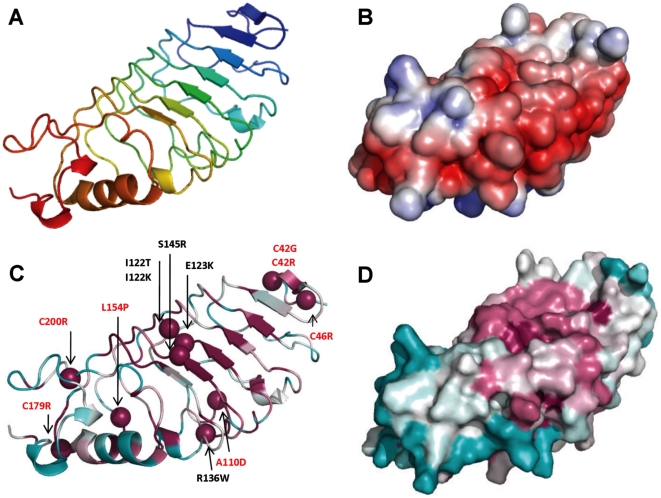
LRR model, structural analysis. A Cartoon of the LRR model coloured from N-terminal (blue) to C-terminal (red); B. Electrostatic surface (negative charge in red and positive charge in blue); C. Position of missense mutations, mutated residues are shown as spheres with structural mutations indicated in red; D. Conserved surface with ConSurf colour code from unconserved (cyan) to strictly conserved (magenta).

The LRR domain was predicted using MANIFOLD. It presents two terminal variable regions, LRR-NT and LRR-CT, reported to have high similarity to those in Nogo-66 receptor (NgR) [51] and four repeats between them. Recently, we presented a preliminary model of the LRR domain based on the NgR structure [7]. Modeling was conducted in two separated steps on the N- and C-termini, which were combined successively. Since the NgR protein has a longer LRR-CT and 8 repeats, the analysis of repeat periodicities with Repetita was performed to identify the correct number of LRR repeats in *LGI1*. The program predicts 4 motifs of 24 amino acids length and the template search selected the structure of the third LRR domain of *Drosophila melanogaster* SLIT (PDB code:1W8AA) [52] as the best template with a 32% sequence identity and the same number of repeats. In this way, the curvature of the LRR domain is more accurately modeled and the residues did not change in relative position as the new model is still based on the alignment from our previous work (Figure 3) [7]. Comparison of conserved residues and secondary structures of hLGI1 and dSLIT revealed many correspondences in the alignment. The alignment was used to build the model, with only two gaps located in the LRR-NT and in the first LRR repeat which were modeled with LOBO. LGI family members and their orthologs differ exactly at these positions. This variability may indicate the presence of a specialized region for the specific LRR domain. Evaluation of model quality by QMEAN indicates that the regions of poor quality are located at the N- and C-terminal portion of the structure (Figure S1). However, the N- and C-terminal caps of the LRR domain present two disulfide bonds (C42–C48 and C46–C55) at LRR-NT and two disulfide bonds (C177–C200 and C179–C221) at LRR-CT which confer stability to the structure. Furthermore, the whole model has good quality as indicated by a QMEAN score reflecting predicted model reliability of 0.6 (range 0,…,1; where 0 is worst and 1 best). As expected, the repeated model core presents all hydrophobic residues forming the consensus sequence in the LRR domain internally buried and polar residues exposed to the solvent (Figure 3). The repeats stack in a parallel arc, allowing to partition the surface into four parts. The concave face, consisting of parallel β-strands, comprises a strong conserved region, while the convex face formed by a tandem arrangement of polyproline II plus β-turns has only localized regions of conservation. We can also distinguish two other surfaces formed by two arrays of loops: the C-terminal side, which contains the loops linking the C-terminal end of the β-strands to the N-termini of the helices, and the C-terminal side, which forms a negative electrostatic surface (Figure 3 and Figure 4). Conserved negatively charged residues in LRR domains have been found involved in specific hydrogen bonds with NH groups of the backbone and considered important for structural integrity [36]. Other solvent-exposed aspartic acid residues have been found to contribute to the twist of the overall LRR structure [53] as in the *Yersinia pestis* cytotoxin YopM [54]. In the LRR domain of LGI1 the negatively charged residues contributing to the negative electrostatic surface are all solvent exposed suggesting that they may be important for protein function.

### Homology modeling of EPTP domain and sequence to structure mapping

**Figure 5:**
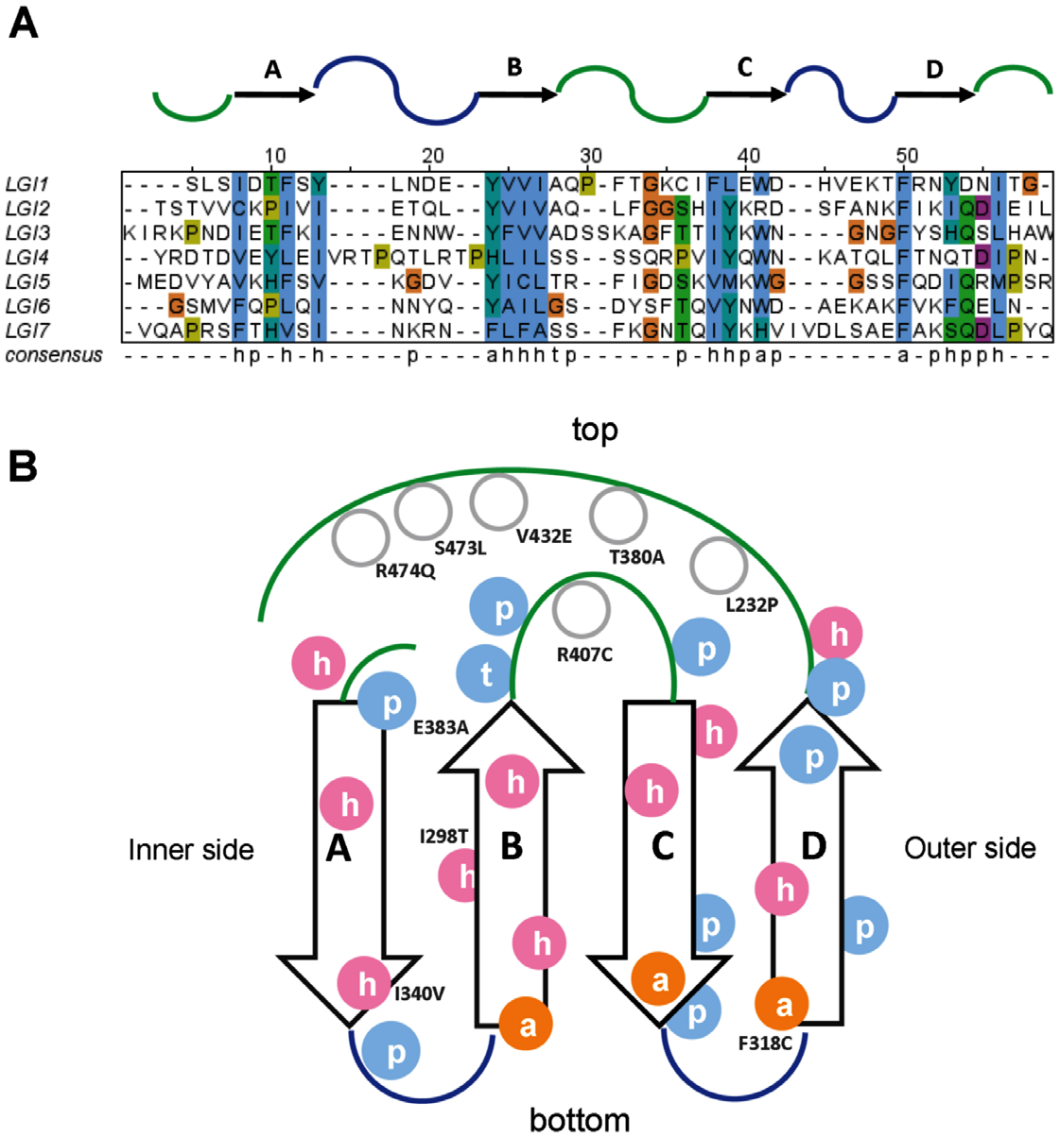
EPTP repeat overview. A. Consensus sequence repeat pattern of EPTP domain. h = hydrophobic residue; p = polar; a = aromatic residue; t = tiny residue. Secondary structure is drawn on the top part of the alignment. Arrows represent β-strands connected by curved lines (loops). Loops forming the top surface are coloured in green, while those forming the bottom surface are coloured in blue. B. Schematic diagram of repetitive structural units in the LGI1 protein. Conserved positions of the consensus pattern are reported on the diagram. Pink and blue spheres indicate buried and exposed residues respectively.

Staub and co-workers [10] proposed that the EPTP repeats could constitute a new class of β-sheet repeats, which fold into a β-propeller structure. The LGI1 β-propeller domain consists of 7 repeats, named EPTP1-7, each comprising a small four-stranded antiparallel β-sheet, whose strands are labeled A to D from N- to C-terminus. Repetita [25] was used to define the boundaries of repeats in the EPTP domain. We built a multiple alignment at the level of single repeats to define the EPTP repeat consensus sequence (Figure 5). In order to classify LGI1 into a specific protein domain family, we searched for the presence of sequence motifs characteristic for different families of β-propellers [55]. The WD motif located at the end of β-strand C is conserved in repeats 1 and 6. In particular, the WD motif at the first repeat is conserved among all LGI proteins. In other blades, tryptophan and aspartic acid are replaced by amino acids with similar biochemical properties (Figure 2). We applied the Metaserver fold recognition method and selected the WD domain structure of human WD repeat protein 5 (WDR5) (PDB code: 2GNQA) as template, which presents a “velcro” closure and ca. 11% sequence identity. In many β-propellers each sequence repeat contains the first three strands of one blade and the last strand of the next. This is apparently also the case for LGI1. We manually curated the alignment between template and LGI1, keeping in consideration the secondary structure prediction. The gaps were closed with LOBO and fell almost all in loops that are longer in LGI1 than WDR5. Evaluation of the model quality, yielding a QMEAN score of 0.4, reveals that the most high quality regions comprise the core of the propeller formed by circular β-sheets, while the loops forming the bottom and top surface show poorer quality (Figure S1). These regions differ more from the template due to the presence of several insertions/deletions. However, we can suppose that the overall model corresponds to the real structure of LGI1, since the protein core is stabilized by hydrophobic interactions. The modeled structure also presents a likely disulfide bridge between Cys260, in the first blade, and Cys286, in the second blade, which would confer further stability to the overall fold.

**Figure 6:**
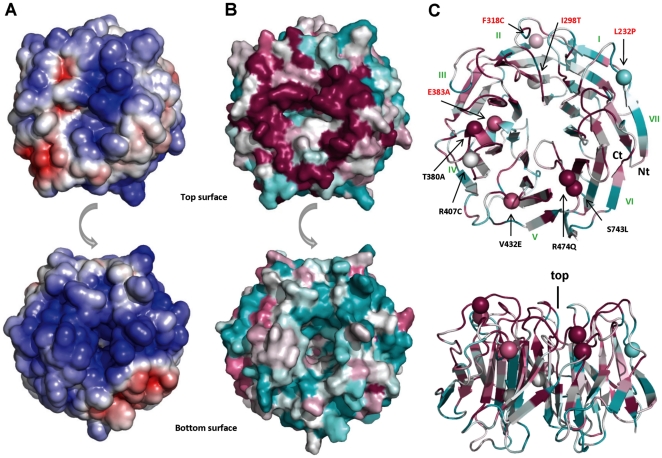
EPTP model, structural analysis. A. Top (up) and bottom (down) view of electrostatic surface of EPTP model (negative charge in red and positive charge in blue); B. Top (up) and bottom (down) view of the conserved surface of EPTP model with ConSurf colouring from unconserved (cyan) to strictly conserved (magenta). C. Cartoon of the EPTP model in top and lateral view with ConSurf colouring. Spheres indicate residues found mutated in ADTLE patients with structural mutations indicated in red.

**Figure 7:**
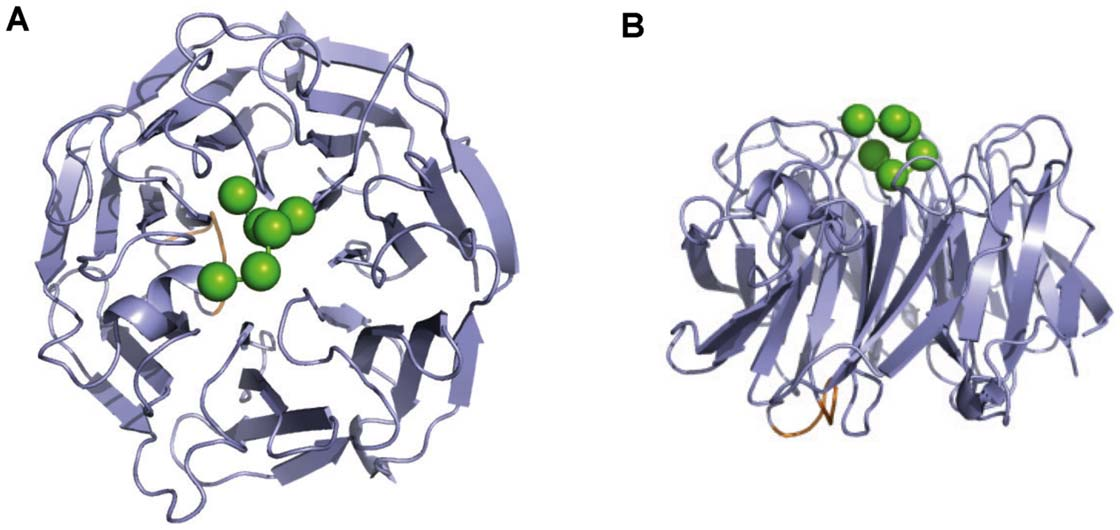
EPTP ligand bindind site. Top (A) and lateral (B) view of the hypothetical peptide binding site on the EPTP model. The position of a hypothetical peptide (green spheres) was obtained by superimposition of the EPTP model with the WDR5 structure (PDB code 3EMH). Note that the insertion specific for LGI1 ( in yellow) maps on the bottom face of the domain.

The LGI1 structural model has been evaluated for both conserved regions and electrostatic surface (Figure 6). Using the alignment of different sequence families retrieved by BLAST, ConSurf does not reveal any particular conserved region. A conserved feature in all modular sheets from different propeller domains is a set of positions with non-polar side chains, generally non solvent accessible, located in the central part of the strands. Since the major determinant for β-propeller assembly is the packing of these residues, amino acids in these positions are free to be replaced by other amino acids with similar biochemical properties [12]. Interestingly, using only sequences of different LGI family members to build the alignment, ConSurf identifies a highly conserved circular region in the top face of the β-propeller. On the bottom face of the protein there are also some conserved sites that correspond to the WD motif and electrostatic surface analysis identifies an extended positively charged region (Figure 6). The top surface is formed by loops connecting strand D of one blade and strand A of the next (DA loops) and loops connecting strand B with strand C in the same blade (BC loops). The bottom surface is formed by loops connecting strand C and D of a blade (CD loops) and loops connecting strand A and B (AB loops) (Figure 5). The alignment of WD repeat sequences allowed the identification of regions of variable length. In some proteins, one or more of these variable regions can be long enough to form an independently folded domain while other insertions form a reverse turn or loop that protrudes from the bottom of the β-propeller [56]. The LGI1 β-propeller has an insertion in the AB loop of the fourth repeat, not presents in paralogous LGI members, that protrudes from the bottom surface (Fig. 2 and 7). This loop may contain a functional motif that contributes to the functional specificity of LGI1.

### Interactions

**Figure 8:**
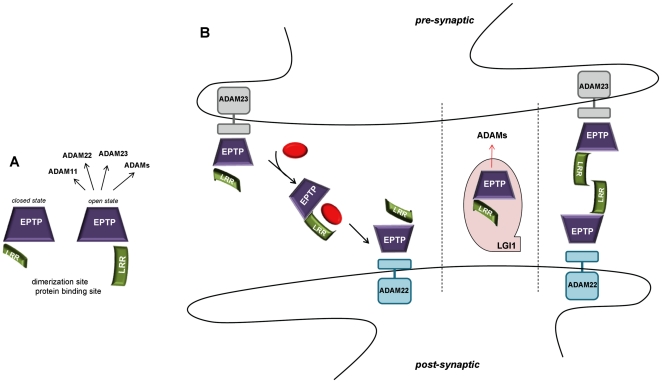
Hypothetical structural assembly and interactions. A. LGI1 is represented as the association of LRR (green arc) and EPTP (violet trapezoid) domains. LGI1 interactions with ADAM proteins likely occur on the top surface of the EPTP domain. B. The two hypothetical ways by which LGI1 could mediate the trans-synaptic interaction between presynaptic ADAM23 and postsynaptic ADAM22.

LGI1 presents two domains that are known to form multi-protein complexes [12], [57]. It is reasonable to suppose that LGI1 mediates interactions between different proteins using different surfaces in the two domains. The first step is to understand how the two domains are arranged together. As they present two surfaces of opposite charge, it can be expected that an attraction between them exists. However, they are not positioned face to face due to the constraint imposed by the short loop connecting them. Instead, if we position the EPTP domain with the top face resting on a plane, the LRR moves laterally above the plane of the bottom surface exposing the conserved β-sheet (concave surface) (Figure 8A). Even if some LRR proteins use alternative surfaces for ligand binding, it is generally thought that the concave surface of the LRR structure contains the ligand-binding site [58]. LGI1 could interact with one protein through the concave LRR interface and with another protein through the top surface of the EPTP domain. It has been previously observed, that the β-propeller structure creates a stable platform that can form complexes reversibly with several proteins, using three potential interaction interfaces: top, bottom and circumference [56], [59].

The top surface appears to be a specialized region for LGI members because it is particularly conserved across them. The superimposition of LGI1 and the complex of WDR5 with its ligand (PDB code: 3EMH) allowed us to map the putative binding site of a ligand on the top surface of the EPTP domain (Figure 7). LGI1 has been shown to bind through the β-propeller domain to both ADAM22, ADAM23 and ADAM11, although with different affinities [60]. On the other hand, *LGI4* is known to interact with ADAM22 [61]. Since the four members of the LGI family have a common phylogenetic origin (Figure 1), it is reasonable to expect that interactions between various components of the LGI and ADAM protein families likely occur through the same, structurally conserved LGI binding site on the top EPTP surface (Figure 8A).

### Role of LGI1 N-Glycosylation

It is well known that the LRR and EPTP domains in LGI1 are N-glycosylated due to their extracellular localization and Sirerol-Piquer et al. [62] demonstrated that N192Q (LRR-CT, conserved across all LGI members), N277Q (conserved across some LGI1 and LGI2 orthologs) and N422Q (only conserved across mammalians) are sites of N-linked glycosylation in LGI1 (Figure 2). Glycosylation could be essential for proper function of the protein since it can dramatically alter surface properties and thereby affect ligand binding. The effect of the potential N-glycosylation sites have been evaluated on the secretion of LGI1 [62]. Compared to a normal protein, the triple mutant was not secreted and secretion of the N192Q mutant was severely attenuated.

To understand the potential role of LGI1 glycosylation we analyzed their distribution over the domain surfaces. In our model, N192 on the LRR domain and N277 and N422 on the EPTP domain are all solvent exposed, confirming the overall correctness of the model. In the LRR domain, the glycosylation site maps to the N terminal side of the LRR-CT portion, while in the EPTP domain, the glycosylation sites map to the β-strand D of the first and fourth blades on the circumference surface. These findings indicate that, while glycosylation modulates the surface properties of LGI1, the putative ligand binding sites are located in non-glycosylated regions.

However, the glycosylation of N192 is supposed to have a mechanistic role. The presence of an oligosaccharide in this position indeed likely interferes with attraction of the charged surfaces present in the two domains, possibly preventing a too close interaction between them. From this point of view, N-linked glycosylation also appears important for correct protein folding.

### In silico analysis of missense mutations

Recently, we have reviewed a total of 25 LGI1 mutations reported in the literature and analyzed their effects on secretion and on the structure using a preliminary model of the LRR domain [7]. Here we present the analysis of all 21 missense mutations found as to date in the LGI1 gene from subjects with familial or sporadic ADLTE, including the recently published p.R407C mutation (Striano *et al.*, in press), the two p.I122T and p.C179R mutations (submitted) and the unpublished p.T380A mutation. Twelve variants affect amino acid residues located in the LRR domain while nine are in the EPTP domain (Figure 4 and Figure 6). The analysis of structural and/or functional effects of these two variant groups has been conducted separately using our models of the LRR and EPTP domains (Table 1). Note that truncating mutations were excluded from our analysis, as no prediction is possible from the structure beyond noting probable protein misfolding.

**Table 1:** Missense mutations overview for the LGI1 protein.

Mutations	dbSNP	Position	Structural/functional effects	Secretion
p.C42R (8)		LRR-NT	Precludes disulfide bridge formation with C48.	NT
p.C42G (8)		LRR-NT	Precludes disulfide bridge formation with C48.	NT
p.C46R (8)	rs104894166	LRR-NT	Precludes disulfide bridge formation with C55.	Negative
p.A110D (8)		LRR2Core	The mutation leads to three neighboring Asp with possible electrostatic repulsion.	Negative
p.I122K (8)	rs119488100	LRR3Core	Insertion of an charged aminoacid (Lys) alters the protein fold.	Negative
p.I122T (8)		LRR3Core	Polar residue inside the hydrophobic core. Possible alteration of the LRR domain fold.	NT
p.E123K (8)		LRR3Concave surface	The mutation alters the electrostatic surface of a potential peptide binding site on LRR domain.	NT
p.R136W (5)	rs119488099	LRR4Convex surface	Arg136 forms a salt bridge with Asp109. The substitution cause the loss of important interactions with neighboring amino acids, leaving tryptophan to protrude from the molecule.	Negative
p.S145R (9)		LRR4Concave surface	The mutation alters the electrostatic surface of a potential peptide binding site on LRR domain.	Negative
p.L154P (6)		LRR4Core	Having two neighboring proline poses a highly destructive condition.	NT
p.C179R (9)		LRR-CT	Prevent the disulfide bridge with C241 causing a misfolding of LRR-CT domain	NT
p.C200R (9)		LRR-CT	Prevent the disulfide bridge with C177 causing a misfolding of LRR-CT domain.	Negative
p.L232P (2)	rs104894167	EPTP7Loop D7-A1 (“Velcro”)	Failure of “velcro” closure. Possible alteration of the protein fold.	Negative
p.I298T (5)		EPTP2βB2	Polar residue inside the hydrophobic core. Possible alteration of the propeller fold.	NT
p.F318C (7)	rs28939075	EPTP2βD2Circumference surface	Position conserved across repeats. Possible alteration of the propeller fold.	Negative
p.T380A (9)		EPTP4Loop D3-A4Top surface	Possible alteration of the functional interactions on the top surface of the propeller.	NT
p.E383A (8)	rs28937874	EPTP4βA4	Loss of contacts with neighboring sheets alter the correct fold of the domain.	Negative
p.R407C (5)		EPTP4Loop B4-C4Top surface	Possible alteration of the functional interactions on the top surface of the propeller.	Secreted
p.V432E (8)		EPTP5Loop D4-A5Top surface	The substitution lead to three negatively charged aminoacids. Possible alteration of the local structural integrity.	NT
p.S473L (9)		EPTP5Loop D5-A6Top surface	Possible alteration of the functional interactions on the top surface of the propeller.	NT
p.R474Q (9)		EPTP5Loop D5-A6Top surface	Possible alteration of the functional interactions on the top surface of the propeller.	NT

The table summarizes conservation degrees from ConSurf (in parenthesis, range 1–9), positions on the protein and predicted structural and functional effects of mutations found in ADTLE patients. For some of these mutants, the effect on protein secretion was previously investigated. For a recent review see [7].

### LRR mutations

Among the twelve variants occurring in the LRR domain, one involves residues on the second LRR repeat, four on the third LRR repeat, two on the fourth LRR repeat and five involve residues at the N- and C-terminus. Some of the considered substitutions mapped at the terminal parts of the LRR domain are of particular interest since they modify conserved cysteine residues flanking the LRR repeats forming disulfide bonds (Figure 2). Substitution of these residues inevitably causes a structural destabilization of the LRR domain. Even if using only protein sequence information, I-Mutant predicts Cys42 and Cys46 as stabilizing, but computational methods are not efficient in predicting protein stability changes due to loss of a disulfide bridge. All LRR variants are predicted to be destabilizing by at least three methods, meaning that all variants could have a negative structural change (Table S1). During initial analysis of LRR variants, we observed that it was possible to distinguish two groups of variants on the basis of their effect on structure or function. The group of structural mutations includes critical mutations of the conserved cysteine residues (p.C42R, p.C42G, p.C46R, C179R and p.C200R), and four mutations of hydrophobic core residues to polar/charged residues (p.A110D, p.I122K, p.I122T, p.L154P). These mutations occur at conserved positions in the LRR repeat alignment having a structural role in folding the LRR domain (Figure 3 and Figure 4). The second group (p.E123K, p.R136W, p.S145R) alter residues located at the protein surface which have a potential to maintain the local structure, the details of which may be crucial for interactions with protein partners. Since all of these mutants lost the ability to be secreted, we hypothesize that a change on the surface, if not causing misfolding, should interfere with the secretion process, e.g. hampering attachment of the protein to the membrane. Evaluation of the electrostatic surface of these three mutants revealed that p.E123K and p.S145R affect the conserved concave surface formed by parallel β-strands of the LRR domain (Figure S2). Variant p.R136W has subtle effects on the electrostatic potential of the convex surface (Figure S2), suggesting this could be another protein binding site.

### EPTP mutations

Nine variants affect the EPTP domain and appear distributed through all repeats without any prevalence for a particular one. All mutations except one (p.S473L) were predicted to be destabilizing by at least two of the computational methods used (Table S1). We also distinguish between structural and functional mutations for the EPTP domain. Three mutations are classified as structural variants (p.I298T, p.F318C, p.E383A), as they affect conserved positions in the repeat alignment and map into the space between the two β-sheets of repeats 2 and 3 (Figures 4 and 5). Indeed, residues forming the consensus sequence of propeller repeats are responsible for the hydrophobic contacts at the inter-sheet cores. It is the packing of these residues that is a major determinant for the assembly of the propeller fold [12]. The variant p.L232P located in the loop between repeats 1 and 7 also has a structural role as it forms part of the Velcro closure conferring stability to the β-propeller (Figure 5).

Interestingly, other variants (p.T380A, p.R407C, p.V432E, p.S473L, p.R474Q) occur at residues located in the DA and BC loops that form the top surface of the β-propeller (Figure 5 and Figure 6). Mutations at the top surface have a potential to interfere with interactions occurring between the β-propeller and molecules such as the known LGI interacting ADAM proteins. In agreement with this, we recently found that the p.R407C mutation does not inhibit protein secretion, probably because it does not perturb the domain fold (Nobile et al., submitted for publication). Therefore, this mutation likely affects the functional properties of the protein binding site on the top surface and manifests its effects extracellularly.

### Functional model

Although a single transmembrane domain was initially predicted in its central part [63], the LGI1 protein does not contain any transmembrane domains and is presumably secreted into the synaptic space [8]. Fukata et al. [17] have recently proposed a model that assigns to LGI1 a role of trans-synaptic adaptor connecting the post-synaptic ADAM22 and the pre-synaptic membrane receptor ADAM23. However, since binding of LGI1 with ADAM proteins is mediated by the EPTP domain [64] and this interaction likely occurs only through the conserved EPTP bottom surface (see above), it is unlikely that LGI1 is capable of interactions with two ADAM proteins simultaneously. Thus, rather than forming a stable link between two ADAM receptors across the synaptic cleft, LGI1 may represent a dynamic link which transports a signal from the pre- to the post-synaptic membrane. In this scenario, binding of a partner protein with the LRR domain removes the EPTP domain from its stable interaction with one ADAM protein and allows the movement of LGI1 to the opposite side of the synapse (Figure 8).

However, it has also been suggested that LGI1 is secreted as an oligomer [14]. Therefore, another possible scenario is that LGI1 could form a dimer, in which the LRR domains of two subunits interact by their concave surfaces connecting two ADAM proteins at opposite sides of the synapse (Figure 8). This supports the experimental findings that demonstrated LGI1 connecting the pre- and postsynaptic machinery through ADAM22 and ADAM23 [17].

The hypothesis concerning LGI1 can also be reasonably extended to other LGI family members. As supported by our phylogenetic analysis and conserved surface residues, binding of ADAM family proteins by LGI is probably a conserved feature. The main difference between LGI1 and other family members appears to be the precise arrangement between the LRR and EPTP domains, as suggested by the presence of a unique insertion on the bottom surface of EPTP in the LGI1 sequences. The effect of this insertion may be a reduced binding affinity for the LRR domain and thus an increased propensity for interaction with other proteins and/or LGI homodimerization. This adaptation could contribute to explain the unique tissue distribution of LGI1 compared to other family members [49].

### Conclusions

An important task of this study was to uncover the relationship between amino acid sequence, 3D structure, and putative functions of the LGI1 protein. Evolutionary sequence analysis revealed the presence of peculiar sequence stretches for each LGI protein, e.g. LGI1 contains a unique insertion on the fourth blade facing the bottom surface of the propeller. Using a structure-based sequence profile we identified a pattern among the structural units and obtained the models which validated several underlying assumptions, including the inward orientation of conserved non-polar residues and solvent exposure of N-glycosylated residues.

The three-dimensional model of LGI1 domains showed how the N- and C-terminal regions are intimately related, revealing a possible mechanism by which LGI1 mediates the trans-synaptic interactions between ADAM proteins. The LGI1 protein contains two conserved binding sites at the concave face of the LRR domain and a circular region on the top surface of the β-propeller domain.

We also evaluated the effect of missense mutations found in ADTLE patients on LGI1 protein and we are able to distinguish between structural and functional mutations, the former potentially causing protein unfolding, while the latter interfere with partner protein interactions. Previously published experiments demonstrated that all but one (p.R407C) tested mutants have a defect on secretion [7] (Striano *et al.*, in press). Thus, we could hypothesize that the secretion-defective mutant proteins are either incorrectly folded or have altered electrostatic surfaces, which could affects LGI1 export. This explains why many LGI1 variants could not be secreted and opens a question about the mechanisms involved in the molecular pathogenesis of the disease. On the other hand, the p.R407C mutation is compatible with secretion, but rather may exert its pathogenic effect by disrupting interactions with ADAM proteins. Other functional mutations may have the same extracellular effect.

Experimental knowledge suggests interactions between LGI1 and ADAM proteins to be mediated by the EPTP domain. We showed that these interactions likely occur through the EPTP top surface. Furthermore, based on the assumption that two protein families usually interact in a similar way, with the same binding site, we predict all four LGI family members to use this interface to interact with different ADAM proteins, albeit with different affinity, in a time and space dependent manner. Finally, we suggest two alternative molecular mechanisms by which LGI1 connects ADAM receptors across the synaptic cleft.

## Supporting Information

Figure S1 **QMEAN model quality evaluation.** The estimated residue error is visualised using a colour gradient from blue (most reliable regions) to red (potentially unreliable regions, estimated error above 3.5 Å).
(TIF)

Figure S2 **Electrostatic potential changes on the LRR surface induced by the E123K, S145R and R136W mutations.**
(TIF)

Table S1 **Analysis of LGI mutations with stability change prediction methods.** The computational predictions were interpreted as stabilizing (S) or destabilizing (D). Stability change prediction is indicated as a ΔΔG value: I-Mutant2.0 (ΔΔG<0 indicates destabilizing variants), Muprot and PoPMuSiC. The protocol used for Eris contains pre-relaxation before calculating the stability change using the flexible-backbone method (ΔΔG>0 indicates destabilizing variants).
(XLS)
