# Supplementary figures and images for: A Computational Model of the LGI1 Protein Suggests a Common Binding Site for ADAM Proteins
Source: PLoS One. 2011 Mar 29;6(3):e18142. doi: 10.1371/journal.pone.0018142 (PMC3066209; doi:10.1371/journal.pone.0018142)

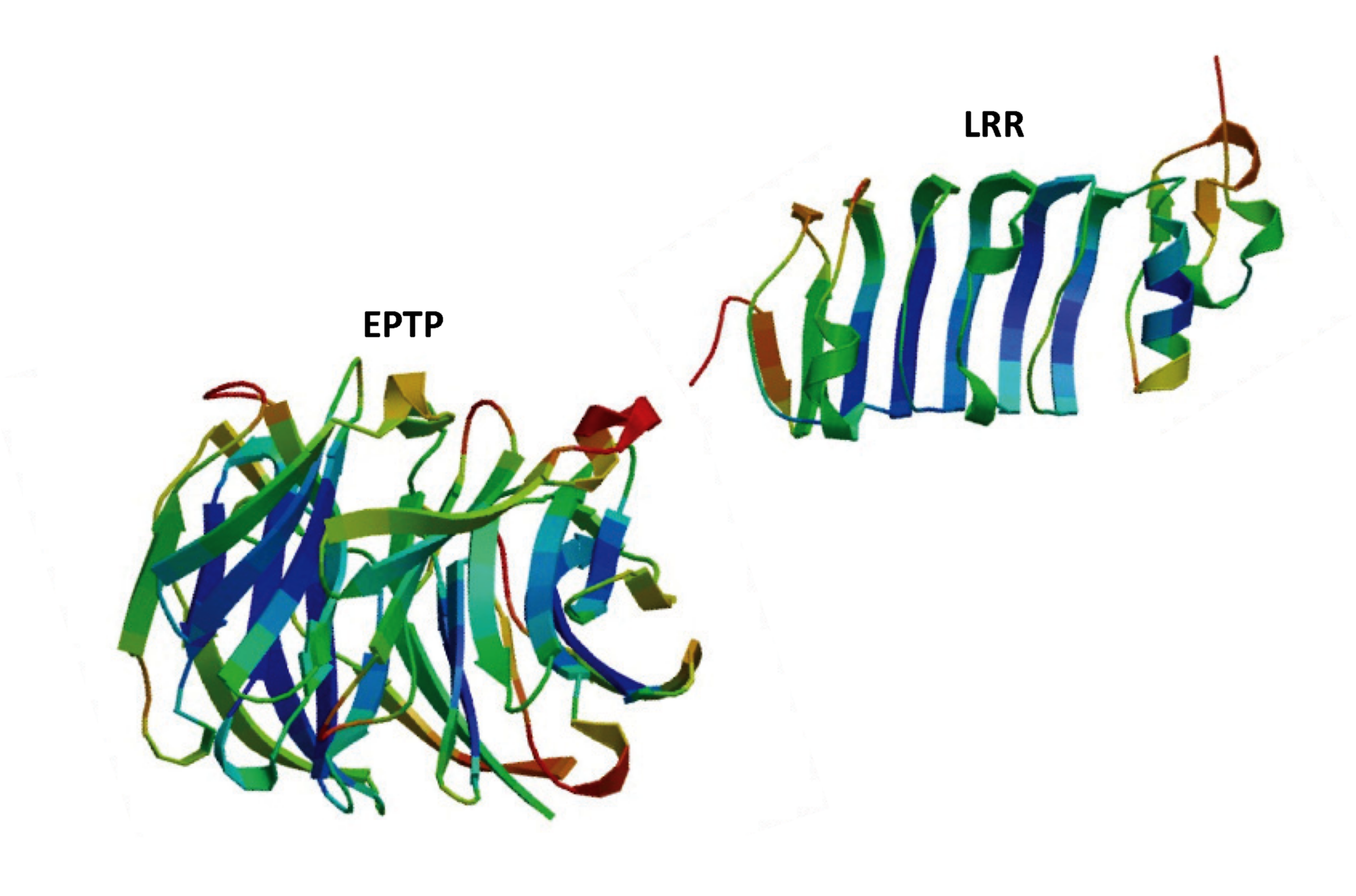

Supplement: Figure S1 — QMEAN model quality evaluation. The estimated residue error is visualised using a colour gradient from blue (most reliable regions) to red (potentially unreliable regions, estimated error above 3.5 Å). (TIF) [file pone.0018142.s001.tif]

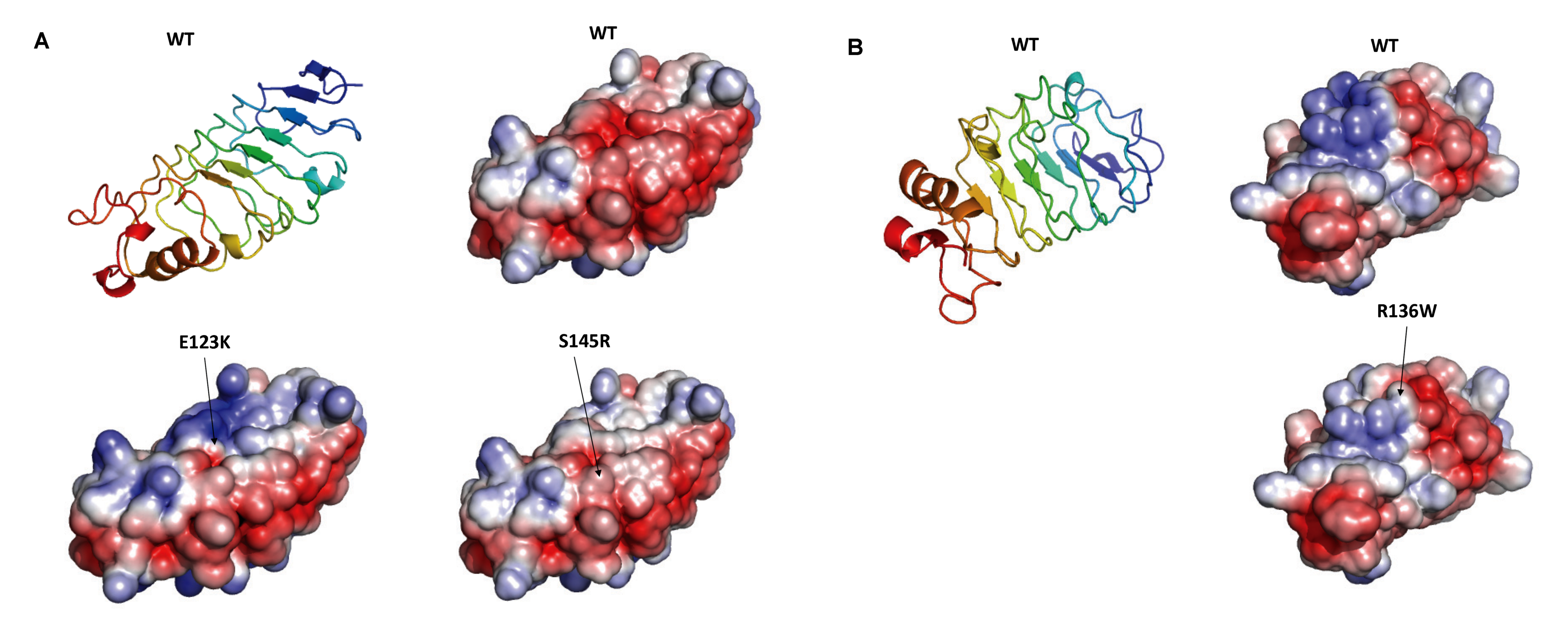

Supplement: Figure S2 — Electrostatic potential changes on the LRR surface induced by the E123K, S145R and R136W mutations. (TIF) [file pone.0018142.s002.tif]
